# Plasma p‐tau181 is associated with brain amyloid deposition and predicts MCI conversion to AD

**DOI:** 10.1002/alz.088213

**Published:** 2025-01-09

**Authors:** Yung‐Shuan Lin

**Affiliations:** ^1^ Taipei Veterans General Hospital, Taipei Taiwan

## Abstract

**Background:**

Plasma phosphorated tau 181 (p‐tau181) was found to change in concordance with amyloid PET and well before tau PET, suggesting that the secretion of p‐tau181 may represent a physiologic reaction to β‐amyloid plaques. Our study aimed to evaluate the relationship between amyloid PET plasma p‐tau181 and whether p‐tau181 predicts amnestic MCI to AD conversion.

**Method:**

We included 98 participants (43 demented and 55 nondemented) who underwent florbetaben amyloid PET scan. Amyloid PET was interpreted by visual assessment based on brain amyloid plaque load score (BAPL). The plasma p‐tau181 level were tested using Simoa assays. Another longitudinal cohort of 37 amnestic MCI patients were tested plasma p‐tau181 level at baseline were followed up at for at least one year. ANOVA and logistic regression were used to evaluate p‐tau181 level between amyloid and cognitive status. Cox regression was used to examine p‐tau181 level and MCI conversion to AD.

**Result:**

Brain amyloid deposition was found in 51% (21/43) of the demented participants, and 11% (6/55) in the nondemented. The plasma p‐tau181 level was significantly higher in the amyloid positive individuals (p<0.001). P‐tau181 alone discriminates amyloid status with an AUC of (0.889, 95% CI 0.826–0.951). In the longitudinal amnestic MCI cohort, 49% (18/37) converted to AD during follow‐up. A P‐tau181 cutoff at 2.545 alone was able to predict conversion where higher Ptau181 has shorter conversion free survival (median survival time in month, 35.0 vs 14.0, p=0.029).

**Conclusion:**

Higher plasma p‐tau181 was associated with brain amyloid deposition and MCI conversion to AD. Plasma p‐tau181 could be a surrogate blood‐based biomarker for amyloid positivity and predicts prognosis in MCI.

